# The In Vivo and In Vitro Effects of *Terminalia bellirica* (Gaertn.) Roxb. Fruit Extract on Testosterone-Induced Hair Loss

**DOI:** 10.4014/jmb.2306.06004

**Published:** 2023-07-21

**Authors:** Min Jeong Woo, Ha Yeong Kang, So Jeong Paik, Hee Jung Choi, Salah Uddin, Sangwoo Lee, Soo-Yong Kim, Sangho Choi, Sung Keun Jung

**Affiliations:** 1 School of Food Science and Biotechnology, Kyungpook National University, Daegu 41566, Republic of Korea; 2 Ethnobotanical Database of Bangladesh (EDB), 7/I, B.F.D.C Road, Tejgaon, Dhaka-1208, Bangladesh; 3 International Biological Material Research Center, Korea Research Institute of Bioscience & Biotechnology, Daejeon 34141, Republic of Korea; 4 Research Institute of Tailored Food Technology, Kyungpook National University, Daegu 41566, Republic of Korea

**Keywords:** Androgenetic alopecia, human follicle dermal papilla cells, *Terminalia bellirica* (Gaertn.) Roxb. fruit extract, testosterone, hair growth

## Abstract

Due to the continuous increase in patients with androgenetic alopecia (AGA) and psychological disorders such as depression and anxiety, the demand for hair loss treatment and effective hair growth materials has increased. *Terminalia bellirica* (Gaertn.) Roxb. (TBE) reportedly exerts anti-inflammatory, hepatoprotective, and antidiabetic effects, among others, but its effects on testosterone (TS)-inhibited hair growth remains unclear. In this study, we evaluated the effects of TBE on TS-induced hair growth regression in human follicle dermal papilla cells (HFDPCs) and C57BL/6 mice. Oral administration of TBE increased TS-induced hair growth retardation. Interestingly, effects were greater when compared with finasteride, a commercial hair loss treatment product. Histological analyses revealed that oral TBE administration increased hair follicles in the dorsal skin of C57BL/6 mice. Additionally, western blotting and immunofluorescence showed that oral TBE administration recovered the TS-induced inhibition of cyclin D1, proliferating cell nuclear antigen (PCNA), and Ki67 expression in vivo. Using in vitro proliferation assays, TBE promoted HFDPC growth, which was suppressed by TS treatment. Thus, TBE may be a promising nutraceutical for hair health as it promoted hair growth in AGA-like in vitro and in vivo models.

## Introduction

Hair has important functions such as protection, thermoregulation, production of pheromones and apocrine sweat, and promotion of social and sexual interactions [1]. Hair comprises two components: hair follicles (HFs) that regulate hair growth and hair shaft that is the visible part of the hair. Hair growth is mediated via interactions between HF dermal papilla cells (HFDPCs) and epithelial cells [2], with HFs undergoing continuous growth (anagen), regression (catagen), and rest phase (telogen) cycles [3]. The anagen phase lasts approximately 3 years on adult scalps and accounts for most of the hair cycle [4]. However, hair loss occurs when hair growth cycles are interrupted, as acceleration from anagen to telogen is induced by different factors, including androgen hormones [5].

Androgenetic alopecia (AGA) is the most common type of hair loss and is characterized by progressive hair follicle miniaturization [6]. AGA occurs in both men and women between the ages of 30 and 50, with global AGA incidences showing a 49.14% increase in 2019 when compared with 1990 [7, 8]. Additionally, AGA causes psychological stress (anxiety and low self-esteem) which lowers the quality of life [9]. AGA is caused by dihydrotestosterone (DHT), a testosterone (TS) metabolite synthesized by 5-α-reductase (5AR). DHT binds to the androgen receptor (AR) in HFDPCs and reduces the anagen phase, leading to hair regression and hair loss [6]. Androgens such as testosterone and DHT bind to the AR and form the DHT-AR complex, inhibiting glycogen synthase kinase-3 beta dephosphorylation and reducing β-catenin expression [10]. Consequently, the expression of DHT-AR-targeted genes, such as transforming growth factor-β (TGF-β) and dickkopf-related protein 1 is increased, suppressing Wnt/β-catenin signaling, which in turn suppresses hair growth factor gene expression (cyclin D1 and c-Myc) [11]. Importantly, natural materials have been developed which restore AGA by regulating AR expression or Wnt/β-catenin signaling [12].

AGA treatments include minoxidil, finasteride (Fina), and dutasteride, which have serios side effects such as sexual dysfunction [13]. Therefore, the demand for safe and efficient natural materials without side effects is warranted. *Terminalia bellirica* (Gaertn.) Roxb. is distributed in India, Bangladesh, and Southeast Asia, and is one of the oldest medicinal herbs [14]. We obtained *Terminalia bellirica* (Gaertn.) Roxb. extract (TBE) from the fruit of its tree. While in vitro and in vivo studies investigating the effects of TBE on anti-inflammatory, hepatoprotective, and antidiabetic processes have been reported [15-17], its effects on TS-induced hair loss remains poorly studied.

We screened several natural materials for restoring AGA, and TBE was selected to examine TS-induced HFDPC growth inhibition and hair growth inhibition in C57BL/6 mice. Oral TBE administration restored TS-induced hair loss in C57BL/6 mice and hair growth marker expression (β-catenin, cyclin D1, and Ki67). Additionally, TBE increased TS-induced proliferative inhibition in HFDPC cells. Based on these preliminary results, we suggest that TBE could be a potent and functional nutraceutical for restoring hair growth.

## Materials and Methods

### Materials

HFDPCs, HFDPC Growth Medium, and growth medium supplementMix (Cat. no. C-26501) were supplied by PromoCell (Germany). Dimethyl sulfoxide (DMSO), Fina, carboxymethylcellulose sodium salt (CMC), and testosterone (TS) were purchased from Sigma Aldrich (USA). Primary antibodies against β-catenin (1:1000), cyclin D1 (1:1000), and proliferating cell nuclear antigen (PCNA) (1:1000) were obtained from Cell Signaling Biotechnology (USA), and Ki67 (1:250) was obtained from Abcam (UK), Santa Cruz (USA) provided the Keratin 15 (1:500) primary antibody.

### TBE Preparation

TBE was obtained from the International Biological Material Research Center, Korea Research Institute of Bioscience and Biotechnology, Daejeon, Korea (FBM 164-010). In our study, 15 g of dried *Terminalia bellirica* (Gaertn.) Roxb. fruit was extracted in 150 ml of 50% ethanol at 50°C for 6 h in a shaking water bath (JS Research, Korea). After filtering through filter paper (GVS, Zola Predosa, Italy), extracts were concentrated in a rotary evaporator (BUCHI, Flawil, Switzerland) and freeze-dried (IlShin Biobase, Korea) at −80°C for 72 h. Dried material was dissolved in DMSO for in vitro studies or distilled water for in vivo studies.

### Cell Culture

We maintained HFDPCs in a growth media with growth medium supplementmix (PromoCell) at 37°C in a 5% CO_2_ atmosphere in a humidified incubator (Germany). HFDPCs were sub-cultured at 70%–80% confluence and cultured up to passage 10.

### Cell Proliferation Assays

HFDPCs were seeded at 2 × 10^4^ cells/ml in 96-well plates. After incubation for 6 h, cells were treated with TBE (6.25–100 μg/ml) for 1 h and then treated with TS (100 μM) for 72 h. Media plus TBE and TS were changed every 24 h. Then, 20 μl of phenazine methosulfate and 3-(4,5-dimethylthiazol-2-yl)-5-(3-carboxymethoxyphenyl)-2-(4-sulphophenyl)-2H-tetrazolium (MTS) (Promega, USA) were added to wells. After a 1 h incubation, absorbance was measured on a microplate reader at 490 nm (Bio-Rad Inc., USA).

### Western Blotting

For western blotting using in vivo samples, separated mouse dorsal skin tissue was lysed using stainless steel beads, lysis buffer (Cell Signaling Biotechnology), and a protease and phosphatase inhibitor cocktail (Thermo Fisher Scientific Inc., USA) in a Precellys 24 dual tissue homogenizer (Bertin, France). Tissue supernatants were centrifuged at 15,000 ×*g* and 4°C for 15 min and quantified using a DC Protein Assay Kit (Bio-Rad Inc.). Proteins were separated using sodium dodecyl sulfate-polyacrylamide gel electrophoresis and then transferred to polyvinylidene fluoride membranes (Millipore, USA). Membranes were then blocked in 5% skim milk in Tris-buffered saline plus 1% Tween-20 (TBST) for 1 h and incubated overnight at 4°C with primary antibodies (Section 2.1). Membranes were washed three times in TBST and a horseradish peroxidase secondary antibody (Thermo Fisher Scientific Inc.) was incubated with membranes for 1 h at room temperature. Protein bands were detected using a chemiluminescence detection kit (Bio-Rad Inc.) and GeneGnome XRQ NPC instrumentation (Syngene, UK). Band intensity was quantified using image J software (National Institutes of Health, USA).

### Animal Studies

**Fig. 1:**
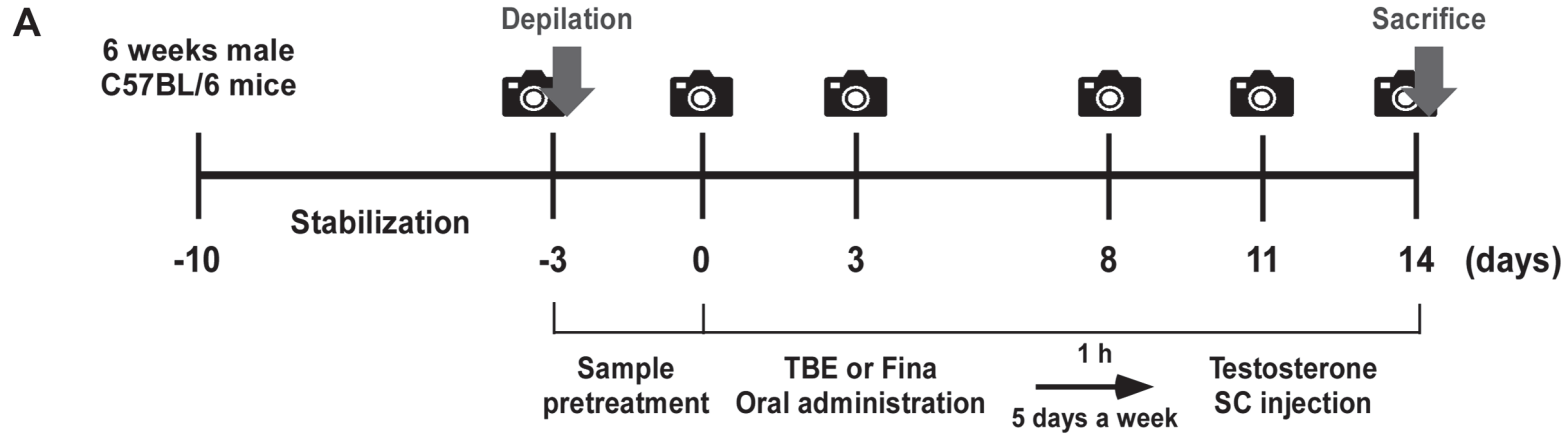
Animal study design evaluating TBE hair growth promoting effects. (**A**) Schematic showing the study protocol. Mice were randomly divided into five groups (*n* = 6/group). Finasteride (Fina) was used as a positive control. TBE (20 and 100 mg/kg) and Fina (20 mg/kg) were orally administered and followed by subcutaneous testosterone (TS) (20 mg/kg) injection 1 h later.

All animals received humane care. The study protocol (KNU-2022-0243) was approved and performed in accordance with guidelines for animal use and care at Kyungpook National University. We purchased 6-week-old male C57BL/6 mice from Samtako, Osan, Korea. Animals were housed in climate-controlled quarters (25°C at 50% humidity) under a 12-h light/12-h dark cycle. Animals were stabilized for 1 week before study commencement and had free access to food and water. Animal study procedures are described (Fig. 1).

Thirty mice were randomly allocated to groups (*n* = 6/group with five groups in total): (1) control group (normal), (2) TS-injected group (TS), (3) TS + TBE low group (20 mg/kg), TS + TBE high group (100 mg/kg), and TS + Fina group (20 mg/kg, positive control). Mouse dorsal skin was carefully shaved using a hair clipper (Babion, Korea) and then depilated with depilatory cream (Beauty Formulas, UK). After oral TBE or Fina administration for 1 h, shaved mice then received a subcutaneous TS injection at 1% CMC in distilled water 5 times per week for 2weeks.

### Immunofluorescence

For in vivo immunofluorescence, Optical Coherence Tomography (OCT) solution (Leica Biosystems Richmond Inc., USA) embedded mouse dorsal skin was cut into 10-μm-thick sections using a Cryostat CM1850 instrument (Leica Biosystems, Germany). After fixation in 4% formaldehyde, tissues were blocked in 5% fetal bovine serum and 0.3% Triton-X 100 in phosphate buffered saline for 1 h 30 min at room temperature. Samples were incubated with specific antibodies overnight at 4°C. Goat anti-mouse or rabbit IgG H&L conjugated to Alexa Fluor 488 or 594 secondary antibodies (Abcam) were incubated with cells or tissue for 1–2 h. Nuclei were counterstained using 4',6-diamidino-2-phenylindole (Abcam). β-catenin expression in HFDPCs, and β-catenin, keratin 15, Ki67, and loricrin expression in C57BL/6 mice was confirmed using fluorescence microscopy (Leica Microsystems, Germany).

### Hematoxylin and Eosin Staining

Frozen 10-μm-thick sections were placed onto microscope slides (Thermo Fisher Scientific Inc.). For histological analyses, tissues were stained with 2.9% hematoxylin solution (Sigma) for 40 min and then dipped in 0.25% eosin solution (Sigma). Tissues were rinsed in cool running water for 5 min after staining steps. Then, sections were sequentially dipped in 50%, 70%, 95% ethanol, and xylene. Stained samples were cover-slipped with mounting solution (Sigma Aldrich). Hair follicle images in mouse dorsal skin were visualized under fluorescence microscopy and analyzed using Leica Application Suite X software (Leica Microsystems).

### Statistical Analysis

Where appropriate, results were calculated as the mean ± standard deviation of repetitions from at least three independent experiments. Student's *t* tests with parametric tests were used for statistical analyses, and between group *p*-values < 0 .05 were considered statistically significant.

## Results

### Oral TBE Administration Restores TS-Induced Hair Growth Inhibition in C57BL/6 Mice

**Fig. 2:**
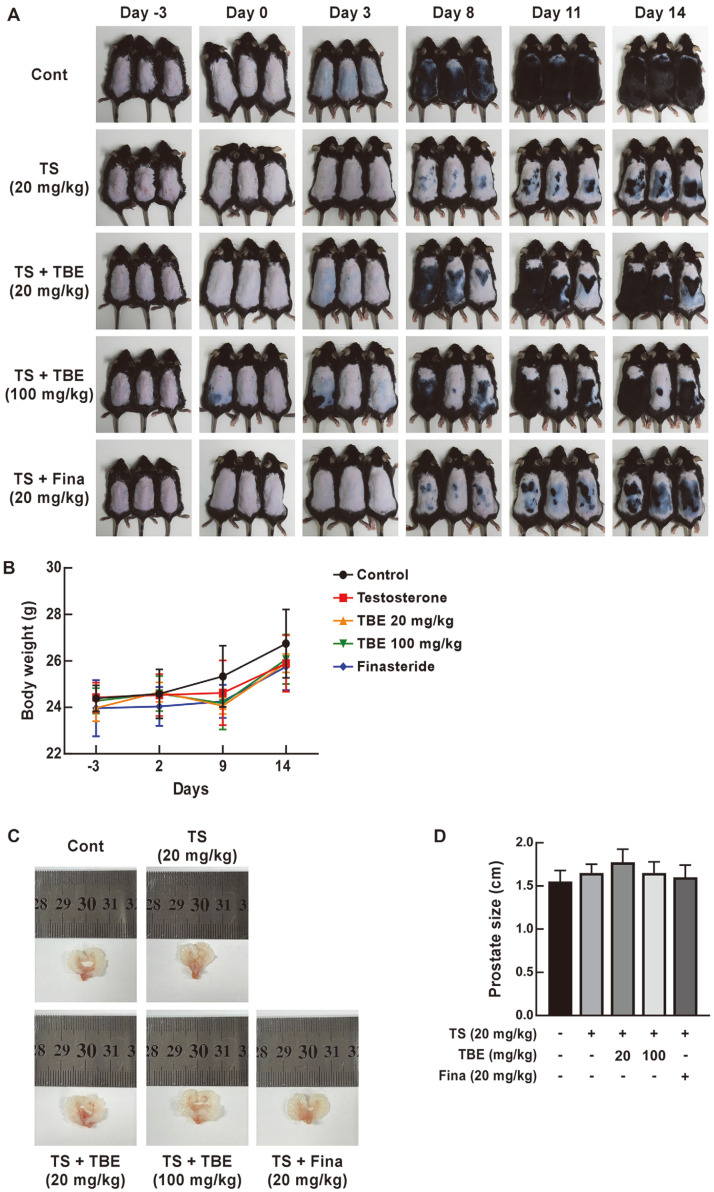
The effects of TBE on TS-induced hair growth inhibition in C57BL/6 mice. (**A**) Dorsal hair regeneration in C57BL/6 mice treated with vehicle (control), TS (20 mg/kg), TS + TBE (20 mg/kg), TS + TBE (100 mg/kg), or TS + Fina (20 mg/kg). (**B**) The effects of TBE or Fina oral administration and TS subcutaneous injection on mouse weight (*n* = 6). Control (●), TS (20 mg/kg, ■), TS + TBE (20mg/kg, ▲), TS + TBE (100 mg/kg, ▼), and TS + Fina (20 mg/kg, ◆). (**C**) and (**D**) Prostate size changes in C57BL/6 mice (*n* = 4).

In C57BL/6 mice, back hair depilation induces a telogen phase which is manifested as pink skin [18]. We confirmed that subcutaneous TS injection for 2 weeks inhibited hair growth in depilated C57BL/6 mice, and TBE and Fina were orally administered to confirm hair growth restoration (Fig. 2A). Oral TBE administration restored TS-induced hair growth inhibition in mice (Fig. 2A). We observed that the dorsal skin in TS-injected mice changed from a telogen stage (pink) to an anagen stage (gray) at day 8 after depilation, while the dorsal skin in control and TBE-injected groups changed from pink to gray at day 3. We observed no significant differences in mouse body weight across groups (Fig. 2B). Furthermore, prostate size changes were observed upon TS subcutaneous injection (male hormone), and no significant differences were identified between TS alone and TS+ TBE groups when compared with controls (Fig. 2C and 2D).

### TBE Increases TS-Induced Suppression of Hair Growth Marker Expression in C57BL/6 Mice

**Fig. 3:**
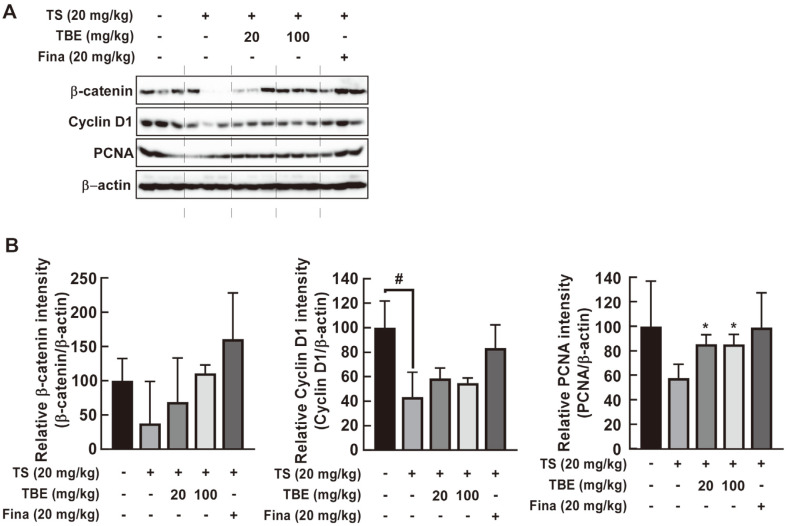
The effects of TBE on TS-induced inhibition of hair growth markers in C57BL/6 mice. (**A**) and (**B**) The effects of TBE on TS-induced inhibition of cyclin D1, β-catenin, and PCNA expression in C57BL/6 mice. Hair growth marker expression in mouse dorsal skin by western blotting. Data are represented by the mean ± standard deviation of three independent experiments. #*p* < 0.05 between control and TS alone groups; *p < 0.05 between TS alone and TS + TBE groups.

Activated β-catenin and increased cyclin D1 and c-Myc gene expression in dermal papilla cells induces hair growth by regulating the cell cycle, with PCNA an important cell proliferation marker [18-20]. Western blotting showed that oral TBE administration significantly increased TS-induced decreases in β-catenin, cyclin D1, and PCNA expression in C57BL/6 mice (Fig. 3A and 3B).

### TBE Significantly Promotes TS-Induced Suppression of HFs and Hair Growth Induction Markers in C57BL/ 6 Mice

**Fig. 4:**
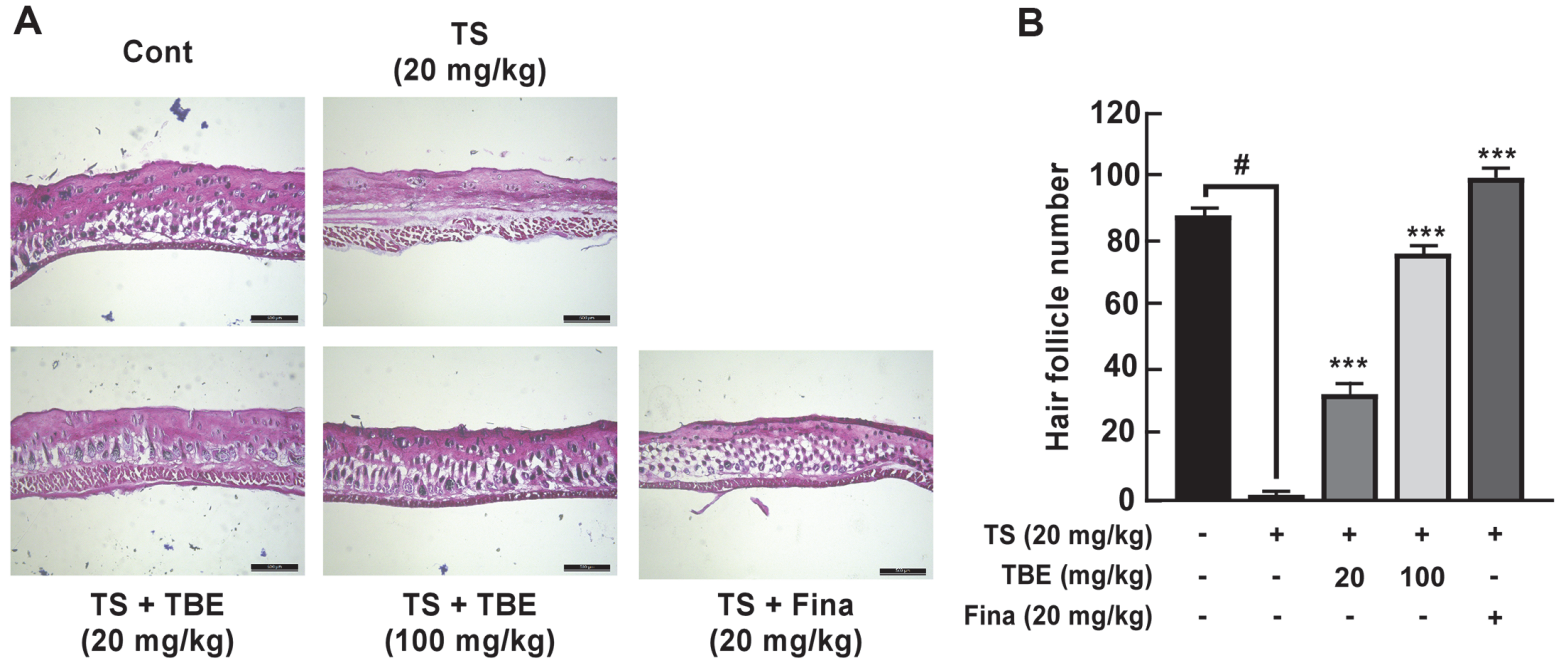
The effects of TBE on TS-induced hair follicle deceases in C57BL/6 mice. (**A**) and (**B**) The histological effects of TBE on TS-induced hair follicle suppression in C57BL/6 mice (H&E staining). Hair follicles (HFs) in mouse dorsal skin were observed under fluorescence microscopy, and HF numbers in the deep subcutaneous layer measured. Data are represented by the mean ± standard deviation of three independent experiments. #*p* < 0.05 between control and TS alone groups; ***p < 0.001 between TS alone and TS + TBE or TS + Fina groups.

**Fig. 5:**
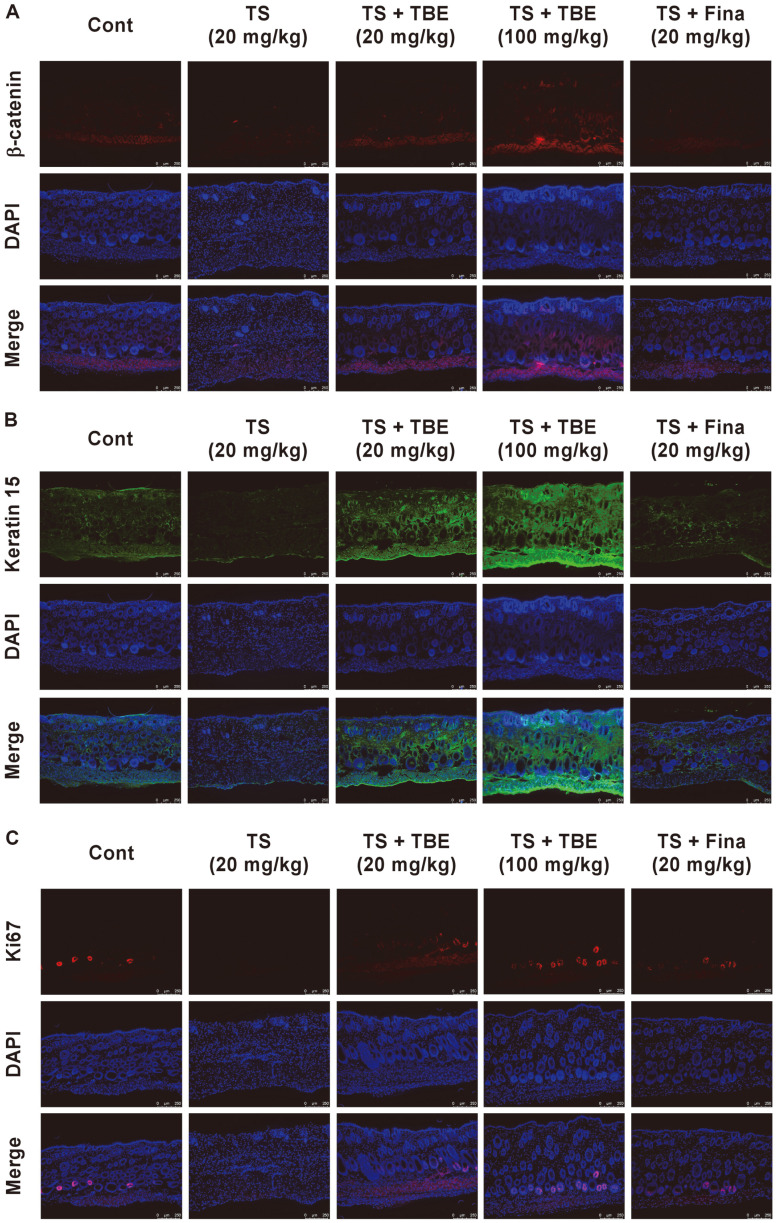
The effects of TBE on TS-induced hair growth marker expression in C57BL/6 mice. (**A–C**) Immunofluorescence showing the effects of TBE on TS-induced β-catenin, Keratin 15, and Ki-67 expression in C57BL/6 mice (Scale bar = 250 μm).

Anagen acceleration of HFs alters the number and size of HFs within the subcutis layer below the dermis [21, 22]. On the other hand, TS promotes the telogen phase and causes hair follicle miniaturization, causing gradual hair thinning and shortening and hair loss [19]. Therefore, we histologically analyzed the effects of TBE on TS-induced HF loss in the subcutaneous layer of the dorsal skin of C57BL/6 mice. Subcutaneous TS injections significantly decreased HFs, whereas oral TBE administration increased TS-induced reductions in HFs in a dose-dependent manner (Fig. 4A and 4B). This result indicated that TBE induced the onset of anagen phase of the hair cycle. Additionally, using immunofluorescence, we confirmed hair growth induction marker expression, including β-catenin, Keratin 15, and Ki67, in mice dorsal skin. Ki67 is expressed in the cell cycle except for G0, and Keratin 15 is a hair growth marker expressed in the outer root sheath in mouse hair [20]. Thus, oral TBE administration increased Keratin 15 and Ki67 expression in the AGA mouse model (Fig. 5A–5C).

### TBE Inhibits TS-Induced HFDPC Proliferative Inhibition

**Fig. 6:**
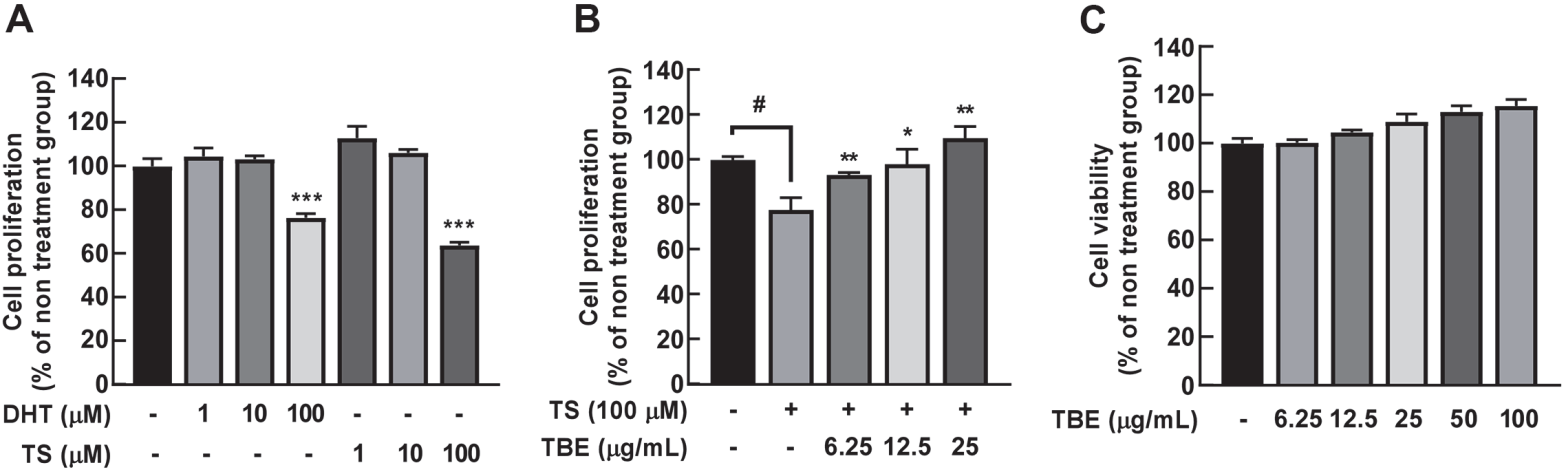
The effects of TBE on TS-induced inhibition of hair follicle dermal papilla cell (HFDPC) proliferation. (**A**) 100 μM TS and DHT were used to inhibit HFDPC proliferation for 72 h. (**B**) TBE restores TS-induced inhibition of proliferation in HFDPCs for 72 h. After cell pretreatment with TBE (6.25, 12.5, and 25 μg/ml) for 1 h, cells were treated with 100 μM TS for 72 h. (**C**) TBE cytotoxicity at 6.25–100 μg/ml in HFDPCs. Cell proliferation and viability were measured by MTS assay. Data are represented by the mean ± standard deviation from three independent experiments. #*p* < 0.05 between control and TS alone groups; *p < 0.05, **p < 0.01 and ***p < 0.001 between TS alone and TS + TBE-treated groups.

We next investigated TBE effects in HFDPCs. We used 100 μM DHT and TS to significantly inhibit HFDPC proliferation (Fig. 6A). We then investigated the effects of TBE on TS-induced inhibition of HFDPC proliferation. TBE treatment induced no cytotoxicity and significantly increased the TS-induced inhibition of HFDPC proliferation (Fig. 6B and 6C).

## Discussion

As hair loss treatments are required by an increasing number of patients with hair loss [8], the hair loss treatment market is expanding. As of 2020, the market was worth approximately $3.8 billion, and it is expected to increase by approximately 1.8-fold by 2027 [23]. AGA is induced by androgen hormones, such as testosterone and DHT. AGA causes social and psychological problems in many patients, as hair loss occurs due to decreased anagen and increased telogen phases durations [24]. In 1951, Hamilton confirmed that AGA occurs due to TS actions [25]. As drugs used for AGA commonly cause side effects [26], the development of safer hair loss restoration materials is warranted [13]. Therefore, we screened for nutraceuticals that induce hair growth and selected TBE as a material with potential hair growth effects.

As the hair cycle based on the age of C57BL/6 mice has been reported in in vivo models for hair loss research [21], we depilated the back skin of mice during hair growth phases to determine if TBE restored TS-induced hair growth inhibition. TBE promoted hair growth more than the positive control (Fina) by inducing growth phases (gray–black) when suppressed by TS. Different signaling pathways, including Wnt/β-catenin [27], TGF-β [28], and sonic hedgehog signaling [29], were previously studied in HFDPCs and C57BL/6 mice. Wnt/β-catenin activation stimulates hair growth by inducing associated protein expression to promote anagen phases [30]. Western blotting data revealed that TBE increased the expression of β-catenin, cyclin D1, and PCNA as well as the expression of Keratin 15 and Ki-67 (immunofluorescence) in C57BL/6 mice. Additionally, HFs on the back skin increased in number owing to oral TBE administration. The DHT-AR complex formed by 5AR conversion constitutively inhibits Wnt/β-catenin signaling [10]. Additionally, the Wnt pathway regulators DKK1 and BMP2/BMP4 can affect β-catenin activity by regulating its stability and ligand–receptor binding [31, 32]. Although we showed that TBE suppressed hair growth marker expression and directly affected HFs, Wnt signaling pathway-related proteins, including the Wnt antagonist DKK1 that induces HF degeneration, HF morphogenesis and regeneration proteins BMP2/BMP4, and Sonic hedgehog, require future investigation. Additionally, our *in vivo* results were reflected in HFDPCs - TBE significantly restored the TS-induced inhibition of HFDPC proliferation.

Overall, TBE alleviated the TS-induced suppression of hair growth in vitro and in vivo. We investigated the effects of TBE on TS-induced inhibition of hair growth in C57BL/6 mice and on HFDPC proliferation. TBE oral administration significantly induced hair growth when compared with Fina-treated C57BL/6 mice. In the dorsal skin of C57BL/6 mice, TBE increased hair growth marker expression (β-catenin, cyclin D1, PCNA, and Ki67) and increased hair follicles. Thus, TBE could function as a nutraceutical for promoting hair regrowth in AGA-like models.
